# Meropenem Continuous Infusion in a Septic Patient with Periprosthetic Infection and End-Stage Renal Disease Undergoing Prolonged Intermittent Renal Replacement Therapy

**DOI:** 10.3390/medicina61010063

**Published:** 2025-01-02

**Authors:** Assiya Kadralinova, Maiya Konkayeva, Serik Dzhandayev, Aidos Konkayev

**Affiliations:** 1 Department of Anesthesiology and Intensive Care, Astana Medical University, Astana 010000, Kazakhstan; konkaev@mail.ru; 2 Department of Anesthesiology and Intensive Care, National Scientific Center of Traumatology and Orthopedics Named After Academician N.D. Batpenov, Astana 010000, Kazakhstan; mkonkaeva@mail.ru; 3 Department of Infectious Diseases and Clinical Epidemiology, Astana Medical University, Astana 010000, Kazakhstan; 4 Department of Ear, Nose, Throat Diseases, Astana Medical University, Astana 010000, Kazakhstan

**Keywords:** infection, meropenem continuous infusion, sepsis, PIRRT, end-stage kidney disease, case report

## Abstract

This case report highlights the use of continuous infusion of meropenem in a 42-year-old septic female patient with periprosthetic infection and end-stage renal disease receiving prolonged intermittent renal replacement therapy (PIRRT). Antibiotic infusion in patients receiving renal replacement therapy has its own peculiarities. There are many studies on the optimal dosing regimen for meropenem in renal dysfunction, but studies on the optimal infusion duration in these patients are limited. The patient was admitted with complaints of wounds, necrosis zones of the right upper limb, restriction of joint movements, and temperature increase up to 38 °C. The patient was treated with a continuous infusion of meropenem 2 g per day receiving renal replacement therapy three times a week (12 h). Also during hospitalization, the patient underwent hip disarticulation and excision of necrotic tissues. The patient was further transferred to a specialized nephrology department for further treatment. We believe that in this clinical case, the use of continuous infusion of meropenem in the complex therapy of sepsis in a patient with CKD undergoing PIRRT sessions helped to lead to clinical improvement in the patient. Further studies are needed.

## 1. Introduction

According to the International Society of Nephrology’s (ISN) 2023 Global Kidney Health Atlas, chronic kidney failure (CKD) affects 850 million people worldwide, representing about 9.5% of the world’s population, and the average mortality rate due to CKD is 2.4% [1]. Septic patients with end-stage renal disease had higher in-hospital and ICU mortality rates than patients without renal disease [2,3].

The uniqueness of this case is that the pharmacokinetics and pharmacodynamics of meropenem in patients with CKD have their peculiarities. Depending on the glomerular filtration rate, patients with CKD on intermittent renal replacement therapy receive a full daily dosage of meropenem. The dose of meropenem in patients on continuous renal replacement therapy is doubled because during prolonged multifiltration, part of meropenem is washed out and the drug’s therapeutic potential decreases. However, the optimal duration of meropenem infusion in patients with renal failure undergoing PIRRT remains unresearched.

In this clinical case, we applied continuous infusion of meropenem to achieve and maintain the drug’s therapeutic concentration in the blood and to reduce its adverse effects on renal tissue, even though meropenem exhibited the least nephrotic effect among carbapenems [4].

## 2. Case Presentation

### 2.1. Patient Information

A 42-year-old woman was admitted to the hospital with symptoms of weakness, dizziness, pain, edema, wounds on her right thigh, endoprosthesis components protruding into the wound, restricted movement in her right hip and knee joints, necrosis on the inner surface of her right leg, and an increase in body temperature up to 38 °C. Moreover, the patient had end-stage renal disease due to tubulointerstitial nephritis, which is defined as GFR < 15 mL/min/1.73 m^2^. The above complaints first appeared 2 months ago, and during the last 5 days, the patient’s condition has worsened and she was admitted to the hospital, directly to ICU.

From the medical history, it is known that the patient has been suffering from right hip joint pain for more than 12 years. As a result of the examination, the patient was found to have a mass in the proximal part of the right hip. Subsequently, the patient underwent surgery in March 2009 for resection of the mass and total right hip replacement. Fibrous dysplasia was diagnosed in the patient based on histopathology of the mass. Thereafter, the patient underwent multiple surgeries for endoprosthesis instability and was treated conservatively for recurrent right hip pain (Pic.1 Timeline). The patient with end-stage renal disease was followed up by a nephrologist and has been receiving hemodialysis sessions three times a week since December 2021.

According to the patient, there is intolerance to brilliant green dye (disinfectant), iodine, adhesive tape with contact dermatitis, and allergy to amikacin with urticaria. There was also a history of allergic reactions to fresh frozen plasma. The heredity, gynecological, and epidemiological history of a patient was without peculiarities.

### 2.2. Clinical Findings

The patient’s general condition at admission was severe, the severity of the condition was due to pain, and intoxication syndromes on the background of end-stage renal disease. The patient’s height was 155 cm and weight was 57 kg. According to the patient, daily diuresis was about 500 mL, and urination was performed independently into the diaper. Clinical examination of the patient’s other organs and systems showed no abnormalities. The patient moved in a wheelchair. Peripheral edema of the upper and lower extremities was also observed in a patient. The right hip’s bandages have a moderate amount of serous-purulent wound discharge embedded in them. On removal of the bandages of the right hip, 4 non-healing wounds of varying sizes were found on the outer surface at the site of postoperative scars. Moreover, the wound in the upper third of the thigh was deep enough that the components of the endoprosthesis were visible from it. The other wounds were covered with scabs. Subsequently, movements in the right hip and knee joints were sharply limited and painful. The right foot was cold to the touch with a weak pulsation on the periphery.

### 2.3. Timeline

**Figure 1:**
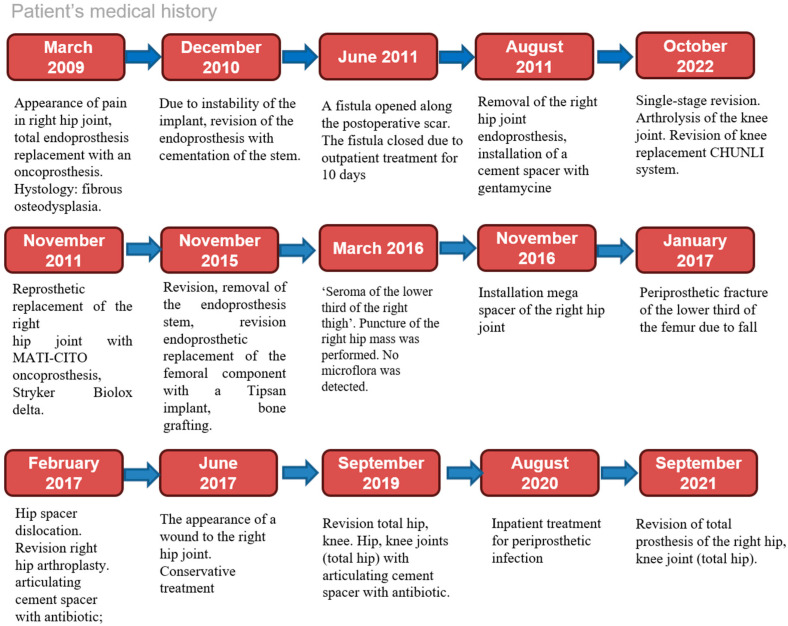
Patient’s medical history.

Figure 1 illustrates the chronological sequence of the patient’s medical history.

### 2.4. Diagnostic Assessment

On the patient’s admission, the results of the complete blood count showed critical values in the form of severe anemia progression (hemoglobin—56 g/L, erythrocytes—1.87 × 10^12^/L), leukocytosis 14.6 × 10^9^/L with a left shift of leukoformula (neutrophilosis—76%, lymphocytopenia—14.4%), and thrombocytosis 505 × 10^9^/L. The erythrocyte sedimentation rate was elevated at 32 mm/h. Coagulation parameters were normal, except for fibrinogen—5.58 g/L. In biochemical blood analysis, the level of C-reactive protein 193.6 g/L reached critical figures, nitrogenous toxins were increased (urea—13.31 mmol/L, creatinine—502 mmol/L), and hypoproteinemia 37.5 g/L and hypokalemia 3.22 mmol/L were observed. From the patient’s words, daily diuresis was about 500 mL, urine analysis showed proteinuria 0.4 g/L, erythrocytes 13–15 cells/μL, and epithelium 5–6 cells/μL. After the transfusion of erythrocytes, metabolic acidosis in venous blood was observed (pH—7.232, PCO2—35.5, BE—12.9 mmol/L). Against the background of continuous infusion of meropenem, a decrease in leukocyte count was observed, and CRP level remained relatively high (leukocytes were 10.6 × 10^9^/L, CRP maximally decreased to 151 g/L). The level of urea was 6.58 mmol/L and creatinine 259 μmol/L.

Microbiological cultures were obtained from the wound, blood, urine, and respiratory samples prior to the administration of meropenem. Further cultures were repeated on the 3rd and 7th day and 1 week before hospital discharge. The patient was admitted with pathogenic flora such as multidrug-resistant Klebsiella Pneumonia and opportunistic polyresistant microorganisms such as *Escherichia coli*, enterococcus, and epidermal staphylococcus (see Table 1).

Following three days of meropenem continuous infusion, the microbiological picture shows the appearance of *Enterococcus* and *Candida*. Furthermore, antifungal medications were added to antibacterial therapy based on culture results. One week after the start of meropenem continuous infusion, we observe an almost primary microbiological picture. Antibiotic therapy was intensified by adding moxifloxacin to meropenem according to antibiotic sensitivity. One week before discharge, cultures showed the presence of nosocomial infection in the form of cultures of *Pseudomonas aeruginosa*.

All required instrumental examination techniques were performed on the patient. A floating thrombus measuring 3.1 cm by 0.63 cm was found in the right jugular vein during the examination of the neck veins. Additionally, the patient had pleural effusions reaching 200–250 mL on the left and 100–150 mL on the right on ultrasound. Abdominal ultrasound showed thickness of the gallbladder wall, cholecystolithiasis, portal hypertension, and hepatosplenomegaly. There were also noticeable alterations in the pancreatic parenchyma. The kidneys’ ultrasound evaluation showed signs of both kidneys’ parenchymal edema as well as changes in the kidneys’ corticomedullary differentiation: 2:1 and 3:1, markers for chronic kidney disease. The X-ray of the right hip and knee shows a total hip and knee joint endoprosthesis. The chest X-ray shows bronchitis.

### 2.5. Therapeutic Intervention

The next morning after admission, a multidisciplinary case conference was held to decide on further medical tactics. It was decided to perform surgery in the amount of disarticulation of the right lower extremity at the level of the hip joint for vital indications after stabilization of the patient’s condition. On the 5th day of hospitalization, a second multidisciplinary meeting was held where the patient’s condition was considered ready for surgery. Furthermore, on the 6th day of hospitalization, the patient underwent the right hip disarticulation surgery. On the next day after surgery, the multidisciplinary meeting was performed, it was decided to intensify antibiotic therapy, taking into account postoperative leukocytosis, and to add moxifloxacin 400 mg twice a day to meropenem according to antibiotic sensitivity.

#### 2.5.1. Respiratory Therapy

The patient received respiratory therapy in the form of oxygen therapy through nasal cannulas from 1 L/min to 6 L/min. The patient also received bronchodilator therapy with budesonide, ipratropium bromide + fenoterol inhalations, and intravenous injection of ambroxol medication.

#### 2.5.2. Antibacterial Therapy

Continuous 24 h infusion therapy with 2 g of meropenem per day via a perfusor was empirically administered for the patient on the first day considering the septic condition of the patient and previous history of cephalosporins intake; 500 mg of meropenem was diluted in 50 mL (10 mg/mL) of NaCl 0.9% solution at an 8.3 mL/h rate to provide a continuous infusion. Following a week of meropenem treatment, antimicrobial therapy intensified with the intravenous addition of 400 mg of moxifloxacin twice daily. During her hospital stay, the patient was also given Imipenem + Cilastatin 500 mg/500 mg, Vancomycine 1000 mg, and Piperacillin + Tazobactam 4 g/0.5 g. Antibiotic therapy was prescribed based on collegial discussions with the participation of a clinical pharmacologist taking into account the clinical and laboratory data of the patient, as well as taking into account the results of cultures and antibiotic sensitivity.

The patient additionally received antifungal therapy—150 mg of fluconazole orally based on the results of crops.

#### 2.5.3. Glucocorticoid Therapy

In addition, the patient was treated with 30 mg of prednisolone or 4 mg of dexamethasone once daily was prescribed during the period of vasopressor support in order to improve the sensitivity of receptors to inotropic agents administered during septic shock.

#### 2.5.4. Anticoagulant Therapy

To prevent thromboembolic complications, the patient received enoxaparin sodium 40 mg subcutaneously one time per day or rivaroxaban 10 mg orally one time per day and heparin 500–1000 ME intravenously or subcutaneously once a day. Acetylsalicylic acid 100 mg orally once was also administered to the patient. Anticoagulant therapy was carried out under the control of hemostasis parameters.

#### 2.5.5. Hemostatic Therapy

Hemostatics, sodium etamsylate 12.5% 2 mL intravenously, and aminocaproic acid 5% 100 mL intravenously were given during the postoperative period and in the event of hypocoagulation to prevent hemorrhagic syndrome.

#### 2.5.6. Pain Management

For pain control, 100 mg of analgin, 100 mg of ketoprofen, 50 mg of tramadol or 20 mg of promedol, even 1% 1.0i/m morphine, and fentanyl 0.005% via a perfusor were used whenever needed.

#### 2.5.7. Liquid Volume Management

The patient also received transfusion therapy using blood components, including freshly frozen plasma, 20% albumin solution, and cleaned erythrocytes to treat anemia and hypoproteinemia, respectively. The transfusion was carried out following a biological pre-testing and the preventative administration of antihistamines due to the history of adverse reactions to fresh frozen plasma.

Every day, the hydrobalance and volemic status were evaluated, and if required, diuresis was induced intravenously with 20 mg of furosemide two to three times each day.

#### 2.5.8. Prolonged Intermittent Renal Replacement Therapy

The patient received renal replacement therapy for 12 h 3 days a week. Epoetin beta 2000 IU p/k was prescribed on hemodialysis days to treat anemia in people with chronic kidney disease.

#### 2.5.9. Nutritional Support

The patient also received nutritional support in the form of intravenous oliclinomel N4 and a diet that restricted their intake of salt and protein due to chronic kidney disease.

#### 2.5.10. Other

Depending on the situation, the patient received sedative therapy, gastroprotective therapy, antiemetic medications (ondansetron), intestinal microbiota improvement (Linex^®^), and correction of water–electrolyte abnormalities. In addition, the patient was administered antihypertensive medication (captopril 25 mg) and vasopressor support of hemodynamics in the form of dopamine, norepinephrine, and epinephrine under blood pressure management.

### 2.6. Surgical Treatment

On the 6th day of hospitalization, the following operation was performed under combined anesthesia: Exarticulation of the right lower extremity at the level of the right hip joint. During the surgery, there was a large blood loss of 800 mL. The patient’s hemodynamics was supported by infusion and transfusion therapy in the volume of saline solution 0.9%—500 mL and washed red blood cells in two doses—270 mL and 280 mL, gelofusine—500 mL. In addition to infusion, the patient’s hemodynamics were assisted with norepinephrine in minimal doses. Then she was transferred to the intensive care unit. On the 33rd day of hospitalization, the patient underwent surgery in the form of primary surgical wound treatment under combined anesthesia. Intraoperative blood loss was 500 mL. During the surgery, the patient received intravenous infusion therapy of sodium chloride 0.9% 1000 mL. Also intraoperatively, 2 doses of washed red blood cells of 280 mL and 290 mL were transfused.

### 2.7. Follow-Up and Outcomes

The patient was discharged from the hospital on the 51st day of hospitalization. At discharge, she was conscious, contactable, adequate, and adynamic. For further treatment, she was transferred to a specialized hospital in the nephrology department.

## 3. Discussion

Patients with CKD on renal replacement therapy have a high susceptibility to the development of infections. Antibiotic therapy for this category of patients is always difficult because there is always a risk of worsening renal pathology. Progression of renal pathology inevitably leads to the death of the patient. There are many recommendations regarding the optimal dosing regimen in patients with renal pathology. For instance, according to the research of Lewis SJ et al., critically sick patients undergoing daily 8 to 10 h PIRRT should take 2 g of meropenem per day (1 g every 12 h or 1 g before and after PIRRT) to get the best pharmacodynamic exposures [5].

According to the population PK model developed for meropenem in critically ill patients with acute renal failure, a meropenem dosage of 0.5 g every 8 h or 1 g every 12 h was appropriate for this population and susceptible bacteria [6]. Meropenem 750 mg every 8 h has been recommended for Asian critically ill patients receiving two different CRRT regimens [7].

Patients with CKD on renal replacement therapy have a higher risk of complications and adverse events compared to patients with a transplanted kidney. Consequently, compared to dialysis patients, total joint arthroplasty is safer and produces superior results for individuals who have had kidney transplants. Thus, it would be preferable to delay total joint arthroplasty in dialysis patients until following renal transplantation [8].

Furthermore, it is still unclear how to administer antibiotic therapy to patients on hemodialysis. According to PK models, PTA can be kept above a 90% threshold by administering a meropenem/vaborbactam infusion either right after HD or at least 2 h before HD starts [9].

According to the study of Keller F. et al., the goal of anti-infective medications with a time-dependent action, including vancomycin, meropenem, and piperacillin, is the high trough. Prolonged infusion is the most effective method of administering such time-dependent medications rather than bolus dosage. The GFR of 30 to 50 mL/min is equivalent to the total filtration rate with continuous renal replacement treatment (CRRT); therefore, many antibiotics do not require a dose reduction during CRRT. To ensure adequate concentrations in the time between the subsequent dosage or dialysis, a fresh loading dose should be administered following intermittent hemodialysis [10].

The investigators O’Jeanson A et al. state in their study that the dosing regimen modeling performed showed that for equivalent doses, the continuous infusion regimen (with loading dose) achieved the pharmacokinetic/pharmacodynamic target for more patients (100% for MIC ≤ 20 mg/L). However, for the treatment of susceptible bacteria (MIC ≤ 2 mg/L), differences in the probability of achieving the target between bolus-like, extended, and continuous infusions were not significant [11].

A limitation of this study is the unavailability to provide therapeutic drug monitoring of meropenem in this clinical case. Additionally, any restrictions that relate to clinical cases are included in the study’s limitations. Such as limited generalizability of the validity of the studies and the impossibility of establishing a cause-and-effect relationship. In our opinion, the antibiotic dosage could be increased to standard doses on dialysis days, as the clearance of meropenem increases during renal replacement therapy. Therefore, a reduction in antibiotic dosage in patients with CKD receiving continuous renal replacement therapy is not necessary [12,13]. It is also possible that less nephrotoxicity of meropenem is expected with continuous infusion, as there is no imposition of shock doses.

According to Jamal JA et al. study, when compared to the minimal concentration (C (min)) seen for individuals receiving bolus infusion (17.0 (15.7–19.8) mg/L; *p* < 0.01), continuous infusion produced a greater meropenem steady-state concentration (26.0 (24.5–41.6) mg/L) [14].

## 4. Conclusions

The use of continuous meropenem infusion in the complex therapy of a septic patient with periprosthetic infection undergoing PIRRT may have resulted in clinical improvement in this case. Further studies in this field are needed.

### Patient Perspective

During and after the meropenem infusion, the patient did not have any negative or unwanted side effects. According to reports, the patient’s health remained unaltered.

## Figures and Tables

**Table 1:** Results of microbiological cultures and antibiotic resistance.

	0 Day Before Meropenem	3rd Day	7th Day	At Discharge
**Blood**	No growth	No growth	No growth	No growth
**Urine**	***Enterococcus faecium*—10^5^** (Azithromycin—R Benzylpenicillin—R Cefoxitin—R Gentamicin—R Levofloxacin—R Sulfamethoxazole and trimethoprim—R) ***Escherichia coli*—10^5^** (Amikacin—R Cefotaxime—R Ceftazidime—R Cefuroxime—R)	***Candida albicans*—10^4^** (Clotrimazole—R Fluconazole—R Itraconazole—R Nystatin—R Voriconazole—R) ***Enterococcus faecalis*—10^5^** (Azithromycin—R Cefoxitin—R Ceftriaxone—R Sulfamethoxazole and trimethoprim—R)	***Enterococcus faecium*—10^5^** (Amikacin—R Azithromycin—R Ciprofloxacin—R Gentamicin—R Imipenem in combination with beta-lactamase inhibitors—R Ticarcillin—R) ***Escherichia coli*—10^5^** (Amikacin—R Cefotaxime—R Ceftazidime—R Gentamicin—R)	***Pseudomonas aeruginosa*—10^4^** (Amoxicillin—R Cefotaxime—R Ciprofloxacin—R Levofloxacin—R Tetracycline—R)
**Respiratory**	***Klebsiella pneumonia*—10^6^** (Cefotaxime—R Ciprofloxacin—R Piperacillin—I Tetracycline—R)	***Candida catenulata*—10^4^** (Clotrimazole—R Fluconazole—R Itraconazole—R Nystatin—R Voriconazole—R)	No growth	***Pseudomonas aeruginosa*—10^5^** (Amikacin—R Cefotaxime—R Cefuroxime—R Ciprofloxacin—R Levofloxacin—R Meropenem—I Tetracycline—R Ticarcillin in combination with beta-lactamase inhibitors (R))
**Wound**	***Staphylococcus epidermidis*—10^3^** (Sulfamethoxazole and trimethoprim—R)	No growth	***Staphylococcus epidermidis*—10^3^**	No growth

## Data Availability

The data presented in this study are available on request from the corresponding author. The data are not publicly available to ensure the confidentiality of the patient’s personal information.
